# Expression of follicle-stimulating hormone receptor by the vascular endothelium in tumor metastases

**DOI:** 10.1186/1471-2407-13-246

**Published:** 2013-05-20

**Authors:** Ahsan Siraj, Virginie Desestret, Martine Antoine, Gaëlle Fromont, Michel Huerre, Marc Sanson, Philippe Camparo, Christophe Pichon, François Planeix, Julie Gonin, Aurelian Radu, Nicolae Ghinea

**Affiliations:** 1 Inserm Equipe Angiogenèse Tumorale, Institut Curie, Centre de Recherche, Département Recherche Translationnelle, 26 rue d’Ulm, 75005, Paris, France; 2 AP-HP, Groupe Hospitalier Pitié-Salpêtrière, Laboratoire de Neuropathologie, R. Escourolle, Paris, France; 3 Département de Pathologie, Hôpital Tenon, Paris, France; 4 Département de Pathologie, CHU de Poitiers, 86000, Poitiers, France; 5 Département de Pathologie, Paris, France; Institut Pasteur, Department Infection et Epidémiologie, Hôpital Curie, Paris, France; 6 Inserm-UMR975; AP-HP, Groupe Hospitalier Pitié-Salpêtrière, Service de Neurologie 2, Paris, France; 7 Centre de Pathologie Amiens, Amiens, France; 8 Icahn School of Medicine at Mount Sinai, New York, NY, USA

**Keywords:** Breast cancer, Colon cancer, Kidney cancer, Lung cancer, Prostate cancer, Endothelial cells, Leiomyosarcoma, Follicle-stimulating hormone receptor, Metastasis, Tumor blood vessels

## Abstract

**Background:**

The Follicle Stimulating Hormone receptor (FSHR) is expressed by the vascular endothelium in a wide range of human tumors. It was not determined however if FSHR is present in metastases which are responsible for the terminal illness.

**Methods:**

We used immunohistochemistry based on a highly FSHR-specific monoclonal antibody to detect FSHR in cancer metastases from 6 major tumor types (lung, breast, prostate, colon, kidney, and leiomyosarcoma ) to 6 frequent locations (bone, liver, lymph node, brain, lung, and pleura) of 209 patients.

**Results:**

In 166 patients examined (79%), FSHR was expressed by blood vessels associated with metastatic tissue. FSHR-positive vessels were present in the interior of the tumors and some few millimeters outside, in the normally appearing tissue. In the interior of the metastases, the density of the FSHR-positive vessels was constant up to 7 mm, the maximum depth available in the analyzed sections. No significant differences were noticed between the density of FSHR-positive vessels inside vs. outside tumors for metastases from lung, breast, colon, and kidney cancers. In contrast, for prostate cancer metastases, the density of FSHR-positive vessels was about 3-fold higher at the exterior of the tumor compared to the interior. Among brain metastases, the density of FSHR-positive vessels was highest in lung and kidney cancer, and lowest in prostate and colon cancer. In metastases of breast cancer to the lung pleura, the percentage of blood vessels expressing FSHR was positively correlated with the progesterone receptor level, but not with either HER-2 or estrogen receptors. In normal tissues corresponding to the host organs for the analyzed metastases, obtained from patients not known to have cancer, FSHR staining was absent, with the exception of approx. 1% of the vessels in non tumoral temporal lobe epilepsy samples.

**Conclusion:**

FSHR is expressed by the endothelium of blood vessels in the majority of metastatic tumors.

## Background

In healthy adult humans, the follicle-stimulating hormone receptor (FSHR) is expressed only in the granulosa cells of the ovary and the Sertoli cells of the testis [1,2]. A minimal expression by the endothelial cells of gonadal blood vessels has also been reported [3,4]. Recently, we have shown that FSHR is selectively expressed on the surface of the blood vessels of a wide range of tumors [5] and we found that FSHR levels in primary renal cell carcinoma tumors correlate strongly with the response of metastatic tumors in the same patients to Sunitinib, an antiangiogenic receptor tyrosine kinase inhibitor [6]. This last observation suggests a link between FSHR expression and angiogenesis in metastatic tumors. However, comprehensive data on FSHR expression in metastases are missing. From a clinical point of view, metastases are more relevant than the primary tumors, because metastases are responsible for the terminal illness, while primary tumors can be surgically removed in most cases. Metastases are the cause of 90% of human cancer deaths [7].

Many of the processes that occur during metastatic tumor growth are similar to the processes in the primary parent tumors, as indicated by similar gene expression profiles of cells in the primary tumors and distant metastases in the same patient [8].

However, significant differences between primary tumors and metastases have been reported regarding protein expression, including cell surface proteins and receptors [9-11], due for instance to disparities between the characteristics of cells that metastasize and cells that remain in the primary tumor [8].

Specific properties of the host tissue could also induce significant differences between the metastatic and the parent tumors. For instance, vascular endothelial cells are known to differ markedly in various organs [12-17]. As a consequence, neoangiogenic processes and the properties of the newly formed blood vessels at distant metastatic sites could in principle display quantitative and qualitative differences in comparison with the parent tumors. This is the case of primary colorectal tumors and their hepatic metastases [18-21].

In the particular case of FSHR expression, a legitimate question is whether it is generally expressed by the endothelium of metastatic tumors, as it is in the primary tumors. The fact that the FSHR is present in all eleven organs from which primary tumors have been analyzed by us [5] indicates that FSHR expression in a tumoral context is a general property of the vascular endothelium in most organs. This observation suggests that vascular FSHR expression occurs in both primary and metastatic tumors, independently of the host tissue.

To determine if this is the case we performed immunohistochemistry experiments. A highly FSHR-specific monoclonal antibody was used to detect FSHR in cancer metastases from 6 major types of cancers (lung, breast, prostate, colon, kidney, and uterine corpus leiomyosarcoma) to 6 frequent locations (liver, lymph node, bone, pleura, lung, and brain).

## Methods

### Tissue specimens

We analyzed metastatic tissues from 209 patients who have been subjected to surgery for removal of the metastatic tumors at the French hospitals: Foch Hospital, Suresnes, Tenon Hospital, Paris, La Salpêtrière Hospital, Paris, Curie Hospital, Paris, and CHU de Poitiers.

The specimens were fixed in 10% formalin for 48 hours and embedded in paraffin. Large sections of 1.5-2.5 cm^2^ were cut from the paraffin blocks corresponding to all types of metastasis, except for breast cancer metastasis to pleura. In the latter case tissue microarrays were constructed using archived formalin-fixed paraffin embedded tissue [22].

All living patients provided written consent to their medical information being used for research purposes. The protocol was approved by the institutional review board or ethics committee at each study site and conducted in accordance with the Helsinki declaration.

### Antibodies

The FSHR-highly specific monoclonal antibody 323 was produced in ascites and purified as described [3]. Goat anti-mouse IgG antibody coupled to peroxidase was purchased from Sigma-Aldrich, Saint Quentin Fallavier, France. Rabbit monoclonal anti-HER2/neu antibody 4B5 was from Ventana, Tucson, Arizona, USA. Monoclonal anti-estrogen receptor antibody 6F11 and monoclonal anti-progesterone receptor antibody 1A6 were from Novocastra Laboratories Ltd, Newcastle, UK. Vectastain Elite ABC peroxidase mouse IgG kit was from Vector Burlingame, CA, USA.

### Chemicals

Sodium borohydride, 3-amino-9-ethylcarbazole (AEC), sodium azide, hydrogen peroxide 30%, goat serum, and haematoxylin Gill solution n°3 were purchased from Sigma-Aldrich, Saint-Quentin Fallavier, France. Diaminobenzidine was from Dako A/S, Glostrup, Denmark. Shandon Immu-Mount medium was obtained from Thermo-Scientific, Asniere sur Seine, France.

### Immunohistochemistry

Sections (5 μm thick) have been cut from the archived paraffin blocks, attached to SuperFrost slides, deparaffinized with toluene, and gradually dehydrated in ethanol. Antigen retrieval was performed by incubating slides at 90°C for 40 min with 10 mM citrate buffer, pH 6. To block endogenous peroxidase activity the sections were incubated with 6% hydrogen peroxide (15 min at RT). Sodium borohydride (10 mg/ml PBS) was used to quench the free aldehyde groups (15 min). Non specific binding of antibodies was blocked by incubating the slides with 2% goat serum in PBS (blocking buffer) for 2 hours at RT. The sections were stained using the anti-FSHR monoclonal antibody FSHR323 (5 μg/ml) [3,5] and subsequently incubated with a goat anti-mouse IgG antibody coupled to horseradish peroxidase (dilution 1:500). The FSHR expression was visualized using AEC [5]. Estrogen receptor, progesterone receptor, and HER-2/neu immunostainings were determined as previously described [23]. The sections were washed in distilled water containing 0.1% sodium azide and counterstained with haematoxylin for 10 sec. The slides were mounted in Shandon Immu-Mount medium.

The sections were analysed using an Olympus BX43 microscope with 20× and 40× objectives and an Olympus SC100 digital camera. Immunohistochemical assessment of FSHR expression was evaluated by six investigators independently (VD, MS, MH, PC, MA, and NG). FSHR-positive vessels were counted using a 20× objective. The density of the FSHR-stained vessels was determined independently by four investigators (AS, AR, CP, and NG) by counting the vessels in fields located inside the tumor, and separately outside the tumor, at a distance of 0-3 mm from the border between the tumor and the normally appearing tissue.

### Controls

Control brain samples consisted of non-tumoral tissue temporal epilepsy (10 patients), tonsillar resection for Chiari malformation (7), lung (3), and pleura (3), all from patients not known to have cancer. Sections from non-tumoral tissue of liver (5 cases), bone (2 cases), and lymph nodes (6 cases) that routinely accompanied surgical tumor samples were used also as negative controls.

## Results and discussion

### FSHR expression in metastases

**Figure 1:**
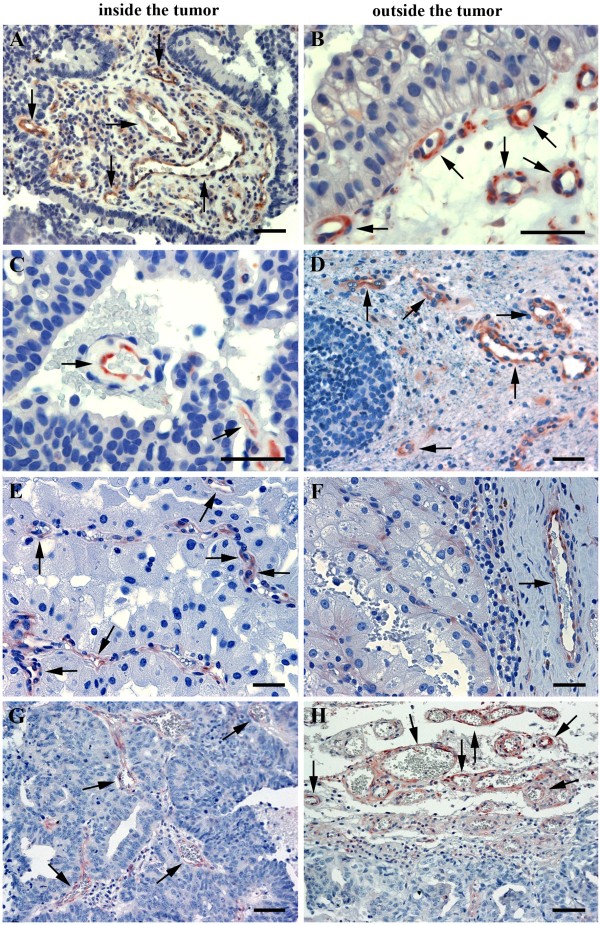
**Expression of FSHR by microvascular endothelial cells in brain metastases of four major cancers.** (**A**,**B**) lung cancer; (**C**,**D**) breast cancer; (**E**,**F**) kidney cancer; (**G**,**H**) prostate cancer. Immunohistochemical analysis was performed on paraffin-embedded sections of human metastatic tissues using the anti-FSHR monoclonal antibody 323, followed by a secondary goat anti-mouse Ig antibody coupled to peroxidase, visualized by the red-brown peroxidase-reaction product. Sections were also stained with hematoxylin. Arrows point to the blood vessels. The scale bar represents 20 μm in all panels.

**Figure 2:**
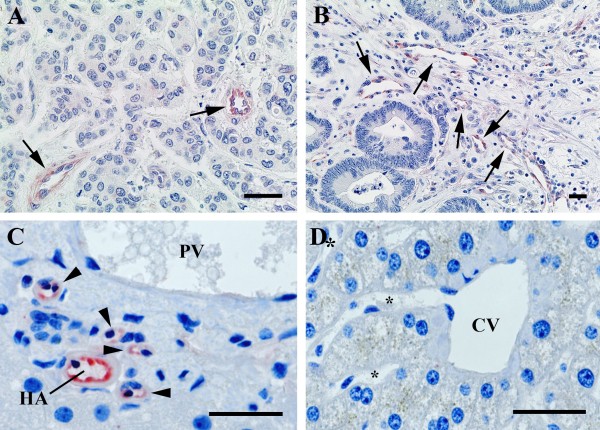
**FSHR-expression in metastases to liver of breast cancer (A) and colon cancer (B).** Immunohistochemical analysis was performed as for the Figure 1. Arrows point to the blood vessels. bc, breast cancer; cc, colon cancer. Panels **C** and **D** show FSHR expression in the normal appearing tissue surrounding metastasis of colon cancer to liver. Endothelial cells of the arterioles and capillaries (arrowheads) derived from the hepatic artery (HA) express FSHR (**C**). The majority of the branches of the portal vein (PV) do not express FSHR. No staining is visible in the central vein (CV) and its associated sinusoids (*) (**D**). The scale bar represents 20 μm in all panels.

**Figure 3:**
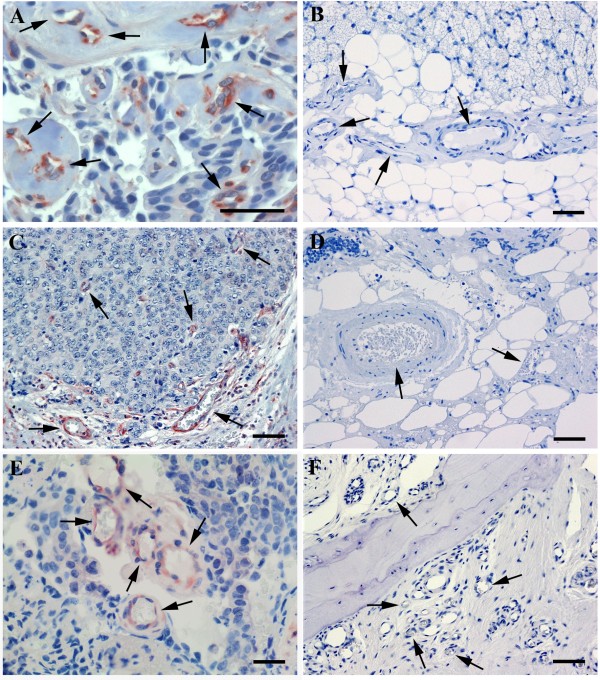
**FSHR-expression in metastases of breast cancer to pleura, and of prostate cancer to lymph node and bone.** Immunohistochemistry was performed as for the preceding figures. Arrows point to blood vessels. Panel **A** shows specimens obtained from patients with breast cancer localized to pleura. Panels **C** and **E** show prostate cancer that metastased to lymph node (**C**) and to bone (**E**). No staining for FSHR is observed in blood vessels of normal tissues in pleura (**B**), lymph node (**D**), and bone (**F**). The scale bar represents 20 μm in all panels.

We investigated FSHR presence in cancer metastases from 6 major types of cancer (lung, breast, prostate, colon, kidney, and leiomyosarcoma) to 6 frequent locations (liver, lymph node, bone, pleura, lung, and brain) of 209 patients (Tables 1 and 2). All tumor types are of epithelial origin, except for uterine corpus leiomyosarcoma which is of mesenchymal origin. Representative images of blood vessels positive for FSHR are shown in Figures 1, 2 and 3. Overall 79.4% (n = 166) of the metastases contained FSHR-positive vessels. No significant differences in the staining intensity were noticed among the cancer types and location of metastases. No staining occurred in tissue sections obtained from patients with metastases if the primary antibody was omitted (not shown).

**Table 1:** Patients’ characteristics

**Cancer**	**Patients**			
	**Female**		**Male**	
	**Number**	Age*	**Number**	Age*
**Lung adenocarcinoma**	**17**	**53 (43-69)**	**29**	**60 (45-79)**
**Colon adenocarcinoma**	**17**	**65 (56-74)**	**17**	**63 (50-65)**
**Prostate adenocarcinoma**	**-**	**-**	**76**	**68 (48-98)**
**Kidney clear cell renal carcinoma**	**2**	**65 (56-74)**	**3**	**63 (50-65)**
**Breast adenocarcinoma**	**42**	**52 (32-69)**	**-**	**-**
**Uterine corpus leiomyosarcoma**	**6**	**60 (19-74)**	**-**	**-**
**Total: 209**	**84**	**-**	**125**	**-**

* Median (range).

**Table 2:** FSHR expression in blood vessels associated with cancer metastases (n = 209)

**Type of cancer**	**Metastases to:**	**Number of cases**	**FSHR immunostained vessels**			
			**No staining**	**Positive staining**	**FSHR-positive vessels/mm**^**2**^	
					**Inside the tumor**	**Outside the tumor**
**Prostate**	**Brain**	**3**	**1 (33%)**	**2 (67%)**	**38.8 ± 13.5**	**117.8 ± 4.5**
	**Lymph node**	**33**	**9 (27%)**	**24 (63%)**	**30.3 ± 17.1**	**74.7 ± 2.8**
	**Bone**	**40**	**26 (65%)**	**14 (35%)**	**33.0 ± 14.4**	**90.3 ± 6.5**
**Breast**	**Brain**	**12**	**0**	**12 (100%)**	**98.4 ± 12.6**	**104.4 ± 4.9**
	**Pleura**	**27**	**0**	**27 (100%**)	**68.7** ± **10.4**	**ND**
	**Liver**	**3**	**0**	**3(100%)**	**100.5** ± **14.4**	**95.2** ± **7.9**
**Kidney**	**Brain**	**5**	**1(20%)**	**4(80%)**	**132.7** ± **11.6**	**108.4** ± **9.9**
**Colon**	**Brain**	**11**	**1(9%)**	**10(91%)**	**87.6** ± **20.1**	**91.1** ± **5.8**
	**Liver**	**23**	**1(4%)**	**22(96%)**	**107.6** ± **20.4**	**112.0** ± **12.0**
**Lung**	**Brain**	**46**	**4(9%)**	**42(91%)**	**115.1** ± **17.8**	**131.1** ± **7.1**
**Uterine corpus leiomyo**-**sarcoma**	**Lung**	**6**	**0**	**6(100%)**	**56.5 **+/-** 9.7**	**97.9** ± **10.9**

ND, not determined.

### FSHR expression in metastases compared with the parent primary tumors

The density of FSHR-positive vessels was determined in 5 primary breast tumors and in 5 metastases to pleura from the corresponding patients. The interior of the metastases had on average 60% (+/-30% (SEM)) more FSHR-positive vessels than primary tumors (p = 0.16, *t*-test, paired, 2-tails). For the two cases of metastases for which adjacent normal tissue was available, the exterior of the metastases had on average 20% more vessels than the exterior of the primary tumors.

### Metastases contain FSHR-positive vessels both inside and outside the tumors

The general characteristic of the FSHR-positive vessels in cancers is that they are located at the periphery of the tumors, in shells that have a thickness of approximately 10 mm (range, 7 to 15) and extend a few millimeters both inside and outside the tumor in the apparently normal tissue. No FSHR-positive vessels were detected in the deeper areas of the tumors [5]. For metastases, FSHR-positive vessels were present some few millimeters outside the tumors, as for the primary cancers. In the interior of the metastases, there was no decrease in the density of the FSHR-positive vessels in the deeper areas, up to 7 mm, the maximum depth available in the analyzed sections (Figure 1 and Table 2). For this reason, all TMA samples of breast cancer to pleura are equally representative, independently of their location (in the periphery or in the interior of the tumor). No significant differences were noticed between the density of FSHR-positive vessels inside vs. outside tumors for metastases from leiomyosarcoma and lung, breast, colon, and kidney cancers. In contrast, for prostate cancer metastases, the density of FSHR-positive vessels was 2.5 to 3-fold higher at the exterior of the tumor compared to the interior, for all three locations (lymph node, bone, and brain) (Table 2; p = 0.001, *t*-test two tails). In a different set of cancer patients previously analyzed [5], in non-metastatic prostate tumors there was no significant difference between the exterior vs. the interior of the tumor.

### FSHR expression as function of the metastatic site

**Figure 4:**
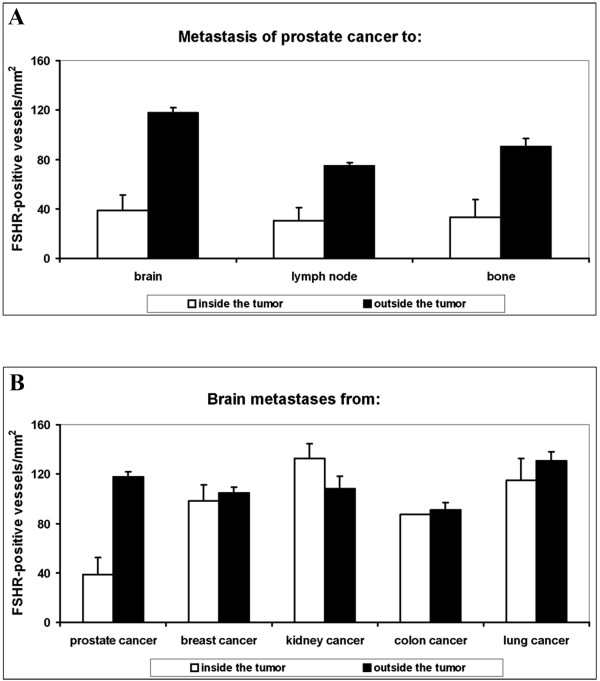
**Density of FSHR-positive vessels in metastases. A**) Metastases of prostate cancer to brain, lymph nodes, and bone. **B**) Metastases to brain of tumors from prostate, breast, kidney, colon, and lung. White and black bars represent vessel densities inside and outside tumors, respectively. Error bars: standard error of the mean.

For some types of cancers, differences in the density of FSHR-positive vessels occur depending on the metastasis site. Thus, metastases of prostate cancer to brain had a higher density of FSHR-positive vessels than metastases to lymph nodes (Figure 4A and Table 2, p = 0.003). No significant differences in the density of FSHR-positive vessels were observed for breast cancer metastases to brain vs. liver (Table 2).

Comparisons of brain metastases from various cancers revealed that the highest density of FSHR-positive vessels occurred for lung and kidney cancer, and the lowest density for prostate and colon cancer (Figure 4B and Table 2).

We investigated previously the vascular FSHR expression in primary tumors of metastatic renal carcinoma patients [6]. After primary tumor removal by surgery, the response of the metastatic lesions to the antiangiogenic drug sunitinib was highly correlated with the level of FSHR presence in the primary tumors. This observation supports a good correlation between the FSHR expression in the primary and the metastatic tumors.

In liver metastases of all types of cancers, the endothelial cells of the arterioles and capillaries derived from the hepatic artery express FSHR in the adjacent tissue located at a distance lower than approx. 1 cm from the tumor (Figure 2C). Approximately 20% of the branches of the portal vein in the normal tissue express FSHR within a distance of some few millimeters from the tumor. No staining was visible in the central veins and its associated venules and sinusoids (Figure 2D). FSHR presence near the bile ducts in the capillaries and branches of the hepatic artery in the tissues adjacent to liver metastases could be related to the observation that liver metastases of colorectal adenocarcinoma sometimes invade the Glisson's triad and grow along the biliary ducts [24,25].

### Correlation of FSHR expression with clinicopathological data

We used the tissue microarrays [22] to analyze by immunohistochemistry the expression of multiple biomarkers in metastases of breast cancer to the lung pleura of 27 patients. We compared the endothelial FSHR expression with the receptor status of three most important markers for breast cancer diagnosis and therapy (estrogen receptor, progesterone receptor, and HER-2/neu). The percentage of blood vessels positive for FSHR was positively correlated with the progesterone receptor level (r = 0.41; n = 25; p = 0.03), but not with the other two receptors. The density of blood vessels did not show a significant correlation with the levels of any of the three receptors.

For the same set of samples, a correlation was detected for the age of the patients with the microvascular density in the tumors, and with the density of FSHR-positive vessels (r = 0.70; n = 17; p = 0.001 in both cases). An increase in the vascular density in elderly patients was recently confirmed for renal cell carcinomas [26].

### FSHR in non tumoral tissue

As controls, we analyzed normal, non tumoral tissues of pleura, lung, liver, bone, lymph nodes, and brain samples of tonsillar resection for Chiari malformation and of cortectomy for temporal epilepsy. The samples revealed absence of FSHR staining (Figure 3B, D, and F), with the exception of a low fraction of vessels stained in the epilepsy samples (on average approx. 1% of the vessels, range 0 - 4.6%).

### FSHR as a tumor endothelial cell marker

Several markers have been described to be preferentially expressed on blood vessels in tumors (e.g., prostate specific membrane antigen, α_v_β_3_-integrin, vascular endothelial growth factor and its receptors, endoglin, etc.) and in the extracellular matrix surrounding newly formed blood vessels (Tenascin-C, matrix metalloproteinases, Robo-4) [27]. Current approaches, which are quite promising in animal models, use integrins as targeted moieties to deliver therapeutics to the tumour vasculature. This was first shown by Hood and his collaborators [28] who used integrin αvβ3-targeted nanoparticles to selectively deliver a mutant Raf1 gene to the tumour vasculature, resulting in apoptosis of endothelial cells and tumour regression.

Integrins do not allow, however, very specific targeting of tumors, and we expect that targeting FSHR will be much more effective. Immunoelectron microscopy experiments with mice that carried LNCaP human xenograft tumors indicate that the FSHR is expressed on the luminal surface of the endothelial cells lining the tumor vessels, in direct contact with the blood [5]. Thus tumor imaging agents, based on FSH or the anti-FSHR-ectodomain antibodies that bind with high affinity to the endothelial FSHR, could be injected in the vasculature and would make visible primary and metastatic tumors located anywhere in the body using magnetic resonance imaging, positron emission tomography, or ultrasound imaging [29]. Our results suggest that FSHR will be able to act as a general target for anti-cancer drugs as well as for agents which destroy or block blood vessels in tumours. Because FSHR is notably absent in most healthy tissues, its use could help minimize the damage that anti-cancer drugs do to surrounding tissue or organs.

## Conclusions

Our data show that FSHR is expressed by the microvasculature of metastatic tumors. This fact strongly increases FSHR potential relevance as a clinical target for cancer imaging and for therapy, especially for tumors that are highly resistant to currently available antiangiogenic treatments.

## Competing interests

The authors declare that they have no competing interests.

## Authors’ contributions

NG, AR conceived and designed the experiments. AS, CP, FP performed the experiments. VD, MA, GF, MS, MH, PC, MS, JG performed histologic analysis. AS, CP, AR, NG analyzed the data. AR, NG wrote the paper. All authors read and approved the final manuscript.

## Pre-publication history

The pre-publication history for this paper can be accessed here:

http://www.biomedcentral.com/1471-2407/13/246/prepub
